# New Insights into the Pathogenesis of Celiac Disease

**DOI:** 10.3389/fmed.2017.00137

**Published:** 2017-08-31

**Authors:** Valli De Re, Raffaella Magris, Renato Cannizzaro

**Affiliations:** ^1^Immunopatologia e Biomarcatori Oncologici/Bio-Proteomics Facility, CRO Aviano National Cancer Institute, Aviano, Italy; ^2^Oncological Gastroenterology, CRO Aviano National Cancer Institute, Aviano, Italy

**Keywords:** celiac disease, B-cell, immunoglobulin, transglutaminase, microbioma

## Abstract

Celiac disease (CD) is an autoimmune and multisystem gluten-related disorder that causes symptoms involving the gastrointestinal tract and other organs. Pathogenesis of CD is only partially known. It had been established that ingestion of gluten proteins present in wheat and other cereals are necessary for the disease and develops in individuals genetically predisposed carrying the DQ2 or DQ8 human leukocyte antigen haplotypes. In this review, we had pay specific attention on the last discoveries regarding the three cellular components mainly involved in the development and maintenance of CD: T-cells, B-cells, and microbioma. All of them had been showed critical for the interaction between inflammatory immune response and gluten peptides. Although the mechanisms of interaction among overall these components are not yet fully understood, recent proteomics and molecular studies had shed some lights in the pathogenic role of tissue transglutaminase 2 in CD and in the alteration of the intestinal barrier function induced by host microbiota.

## Introduction

Celiac disease (CD) is characterized by small intestinal mucosal injury and nutrient malabsorption (1). Studies of stored serum showed an increase in CD prevalence from fourfold to fivefold over the past 50 years (2). The geographic epidemiological distribution of CD along a north–south and west–east gradient in Europe related to socioeconomic status and observations from population migration suggests an environmental impact driving the increased incidence of CD but this topic is still more debated (3, 4). Nowadays, we know that CD results not only in variable degrees of small intestinal inflammation but also on a wide range of both gastrointestinal and extra-intestinal manifestations, and sometimes even in asymptomatic patients (5). The more common extra-intestinal symptoms include abnormal liver enzymes, arthralgia/arthritis, alopecia, fatigue, headache, anemia, mouth sores, muscle aches, depression, rashes, neuropathy, short stature, delayed puberty, osteoporosis, and infertility.

Celiac disease clinical identification is based on histological evaluation of the small intestine biopsies. Serologic assays, such as immunoglobulin (Ig)-A tissue transglutaminase and endomysial antibody (EMA; IgA), may be useful to support the diagnosis (6).

It is well accepted that CD represents a multifactorial etiology with a strong genetic background that involves both human leukocyte antigen (HLA) and non-HLA genes. In particular, it is widely known that CD occurs in genetically predisposed individuals carrying the DQ2 and DQ8 HLA haplotypes, a condition that is necessary but not sufficient for CD development since these molecules are widely (25–40%) distributed among the population but not all the individuals had the disease (7). DQ2 and DQ8 molecules serve as restriction elements for gluten-specific CD4^+^ T lymphocytes, but it is not sufficient and it is a necessary alteration in the immune response to cause the chronic small intestinal inflammation. As a consequence, HLA typing may be used to search for individuals with an increased risk of developing CD and it is particularly relevant: when pathology of the small intestine is equivocal, when serological testing is consistent with CD but villous atrophy is absent, when a gluten-free diet is being considered in the absence of biopsy-proven CD, or when a first-degree relative has been diagnosed with CD. Determining genetic susceptibility can avoid unnecessary small intestine biopsy, continual serologic testing, and initiation of a gluten-free diet, especially in individuals in high-risk groups, such as first-degree relatives of patients with CD, patients with insulin-dependent diabetes mellitus, with thyroiditis, with Down, Turner or Williams syndrome, with selective IgA deficiency or with an unexplained iron deficiency anemia (8, 9).

It is clear that ingestion of gluten protein is a prerequisite of disease development, resulting in villous flattening or crypt hyperplasia (7). Traditionally, gluten is a name of wheat proteins only, but gluten is now increasingly used as a term to denote proline- and glutamine (Gln)-rich proteins made by a class of grasses called *triticeae*, which mainly includes wheat, barley, and rye. A large number of studies have been produced and are ongoing regarding this theme as we better develop in a subsequent chapter. A gluten-free diet reverses many disease manifestations, but it is not sufficient in a minority of patients with refractory CD. Since patients having a refractory CD and unusual clonal T-cell population (type II refractory CD) are at risk for a particularly aggressive form of non-Hodgkin’s lymphoma the follow-up of these patients is particularly important and recommended (10).

Tissue transglutaminase 2 (TG2), the target of IgA autoantibodies, was also demonstrated to play a key role in the pathogenesis of CD. Autoantibody reactivity to TG2 is a specific and sensitive marker highly useful in aiding diagnosis and follow-up of CD, but the mechanism by which TG2 becomes the main autoantigen in CD is not yet understood (11) and we will discuss this point subsequently.

Finally, since recent evidence based on global epidemiological and scientific studies has suggested that the risk of some conditions, such as obesity, inflammatory bowel disease, cancer, and also some autoimmune diseases, may be influenced by the microbioma (12) we will discuss the role of microbiome in CD pathogenesis.

## Gluten-Specific CD4^+^ T-Cells: High Producer of IFNγ and Low Producer of Bach2

It is widely accepted that fragments of gliadin, one of the main component of gluten, present in food pass through the epithelial barrier of the small intestine and enter the lamina propria where they are deaminated by tissue TG2 and then linked to specific HLA-class II (DQ2 or DQ8) molecules. The presence of T-cell receptors (TCRs) specific for the recognition of HLA-gliadin complexes lead to the IFNγ and interleukin 21 production, which favors the CD8^+^ T-cell cytotoxic activity accountable for the intestinal mucosal damage.

**Figure 1:**
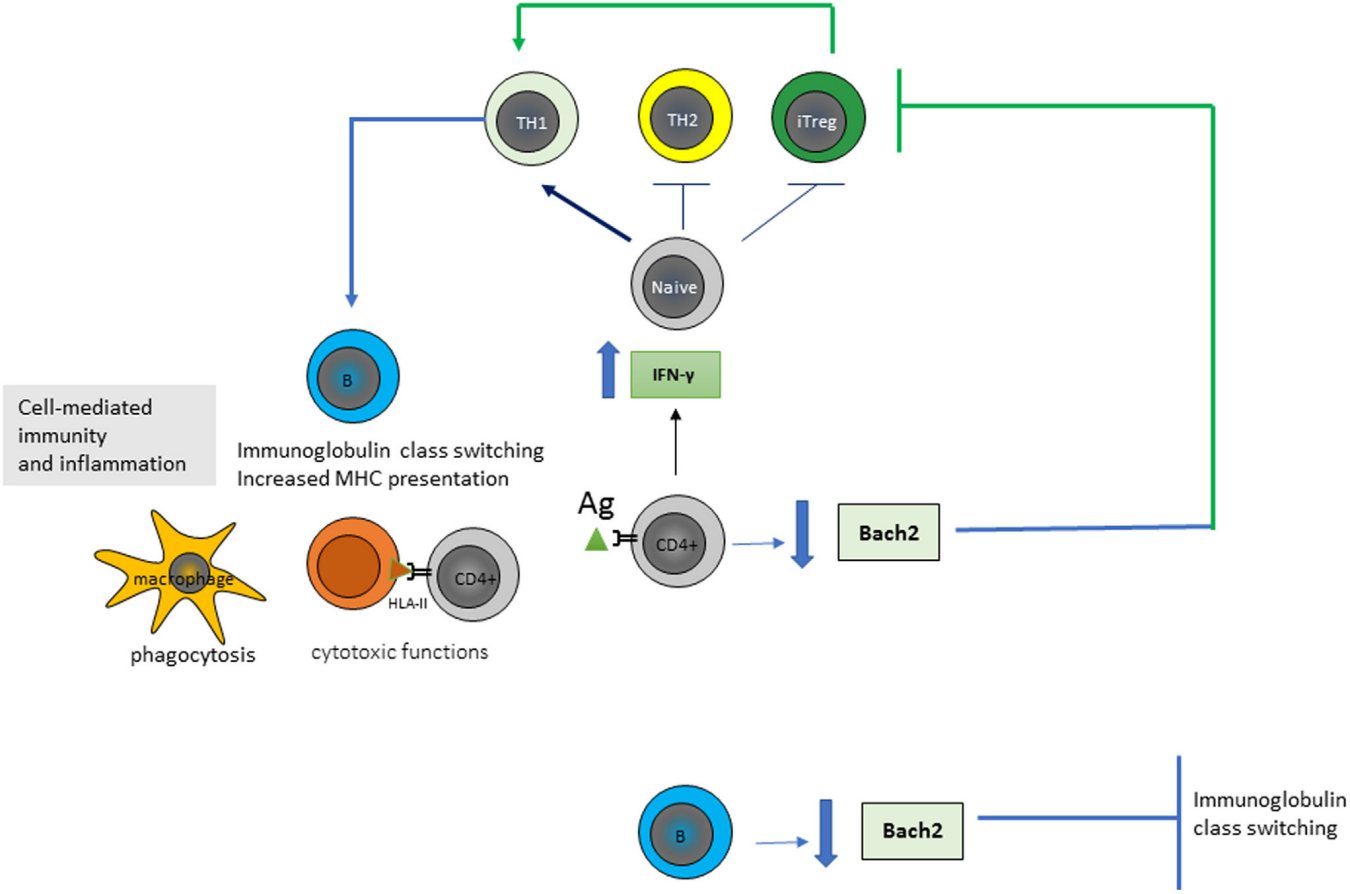
Gluten-specific CD4^+^ T-cells of patients affected by Celiac disease show an high production of interferon-gamma (IFN-γ) and a low production of Bach2. CD4^+^ T-cells proliferate and differentiate into various subtypes in response to antigen (Ag) stimulation and their microenvironment. IFN-γ is known to promote the differentiation of T-helper 1 (TH1) cells, inhibit the TH2 immune response and Treg survival, and activate the phagocytosis of macrophages, B-cell immunoglobulin class switching, and presentation of major histocompatibility complexes (MHCs). Bach2 expression is crucial for the development of Treg and for the germinal center formation, somatic hypermutation and class-switch recombination of immunoglobulins in B-cells; thus is an important key regulator in the maintenance of immune homeostasis.

More recently, there was directed attention toward alterations characterizing the T-helper 1 (TH1) CD4^+^ T-cell population in CD. Tanscriptome next-generation RNA-sequencing (RNA-seq) analysis, based on new next-generation sequencing technologies, confirmed in CD the potential importance of the proinflammatory cytokine IFNγ, which displayed a ~25-fold increased expression in CD cases compared to controls (13). Classically, IFN-γ is known to promote the CD4^+^ T-cells differentiation toward a profile of TH1-cytokines type, while it is known to inhibit the TH2 immune response and the regulatory T-cell survival. TH1-cells are known to develop cell-mediated immunity by enhancing and maintaining response of CD8^+^ (cytotoxic) T cells and to activate the phagocyte-dependent inflammatory status. Moreover, CD4^+^ T cells have direct cytotoxic functions by secretion of cytotoxic granules containing granzyme B and perforin and by a direct kill of target cells in an antigen (Ag)-HLA-class II fashion upon direct contact. Ag-presenting cells (i.e., dendritic cells or B-cells) present peptides derived from both exogenous Ags phagocytosed and processed in the endosomes and from endogenous processing *via* cell autophagy. TH1-cells also were known to promote B-cell Ig class switch and presentation of HLA (Figure 1).

Tanscriptome RNA-seq analysis of CD4^+^ T-cells isolated from patients with CD highlighted additional information regarding the pivotal role of CD4^+^ T-cells in CD discovering that expression of the transcriptional repressor BACH2 gene was specifically reduced in these cells and resulted a key regulator of CD4^+^ T-cell senescence and cytokine homeostasis (14). BACH2 is a transcription factor of the basic leucine zipper family, which first has been identified as crucial for the germinal center formation, somatic hypermutation, and class-switch recombination of Igs in B-cells. It was now demonstrated that BACH2 gene expression was critical not only for B-cells but also for the maintenance of cellular immune homeostasis of T-cells to counterstain excessive immune activation and inflammation (14). Indeed, studies regarding its function in T-cell population had demonstrated that BACH2 regulated transcriptional repression of genes associated with differentiation of CD4^+^ T-cell into TH1, TH2, and Treg and that its absence during Treg polarization resulted in an ineffective formation of Treg and, thus, in an inappropriate suppression of inflammation (Figure 1).

Thus, today a network mainly involving a significant increase in IFNγ and a significant reduction in BACH2 expression are known to drive the CD4^+^ TH1 cell-mediated inflammatory status associated with CD. However factors impacting on the pathological expression of these genes remain to be deciphered and they could be dependent on signals directly deriving from CD4^+^ T-cells as well as from other cells. Furthermore, considering the key role of BACH2 also in directing B-cell lineage and adaptive immunity, there is a need for further researches to understand the mechanism of BACH2 regulation and its action in mediating immune response in physiological–pathological status.

## TG2, a Generator of Immune T-Cell Epitopes and a Target of Autoantibodies

**Figure 2:**
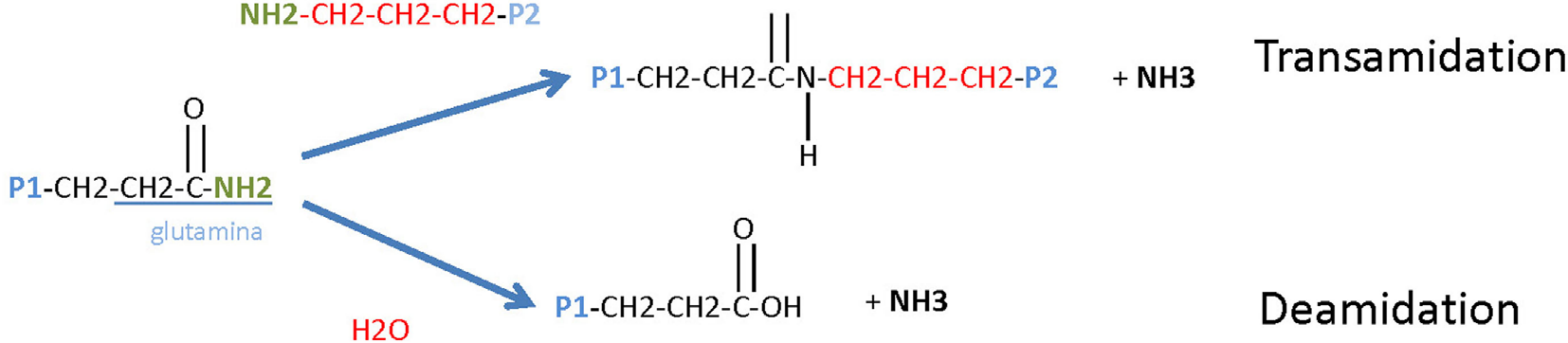
Tissue transglutaminase 2 (TG2) enzymatic activity. TG2 is a ubiquitously expressed member of the TG family of enzymes that all catalyze Ca^2+^-dependent protein deamidation or transamidation (cross-linking) of glutamine residues.

Tissue transglutaminase 2 was found to play a key role in the pathogenesis of CD and is the target for TG2 autoantibodies, a marker for the diagnosis and the follow-up of CD. TG2 is a calcium-dependent enzyme, which catalyzes transamidation or deamidation of Gln residues in protein and peptide substrates. By cross-linking protein on Gln residues, TG2 creates intramolecular and intermolecular bound that are highly resistant to protein degradation (Figure 2). TG2 is the most prevalent member of the mammalian transglutaminase family, with abundant intracellular and extracellular expression in most organs. Intracellular TG2 is enzymatically inactive and for reasons that are not understood, TG2 is transported extracellularly, where, it become enzymatically active. In the extracellular space, TG2 binds to matrix proteins, particularly fibronectin, playing a role in cell adhesion, extracellular matrix stabilization, wound healing, receptor signaling, cellular proliferation, and cellular motility. TG2 expression is regulated by several post-translational mechanisms, but the mechanism of its regulation is not completely understood.

It is fully demonstrated that TG2 is upregulated in the mucosa of the small intestine during active CD. Moreover, it is demonstrated that IFN-γ, is the most potent inducer of TG2 and that its synergism with TNF-α contributed to exacerbate CD (15). However, a high TG2 expression is not unique to CD and anti-TG antibodies are often observed in other inflammatory conditions, such as inflammatory bowel diseases, such as Crohn’s disease and ulcerative disease, and juvenile diabetes.

Tissue transglutaminase 2 is particularly notable for being the autoantigen that may contribute to intensify the inflammatory process in CD patients by destruction of villous extracellular matrix and as a target for intestinal villous epithelial cell destruction (11). By its transamidation capacity, TG2 is known to cross-link gliadin peptides to itself resulting in the generation of TG2-gliadin complexes. Gliadin is a favored dietary substrate for TG2 because of the presence of many proline- and Gln-rich sites. The TG2/gliadin complexes might provide the generation of the TG2 autoantibodies, but the exact mechanism by which TG2 becomes the main autoantigen in CD has not been settled. In this section, we aim to summarizing the last hypothesis for the generation of TG2 autoantibodies in CD.

**Figure 3:**
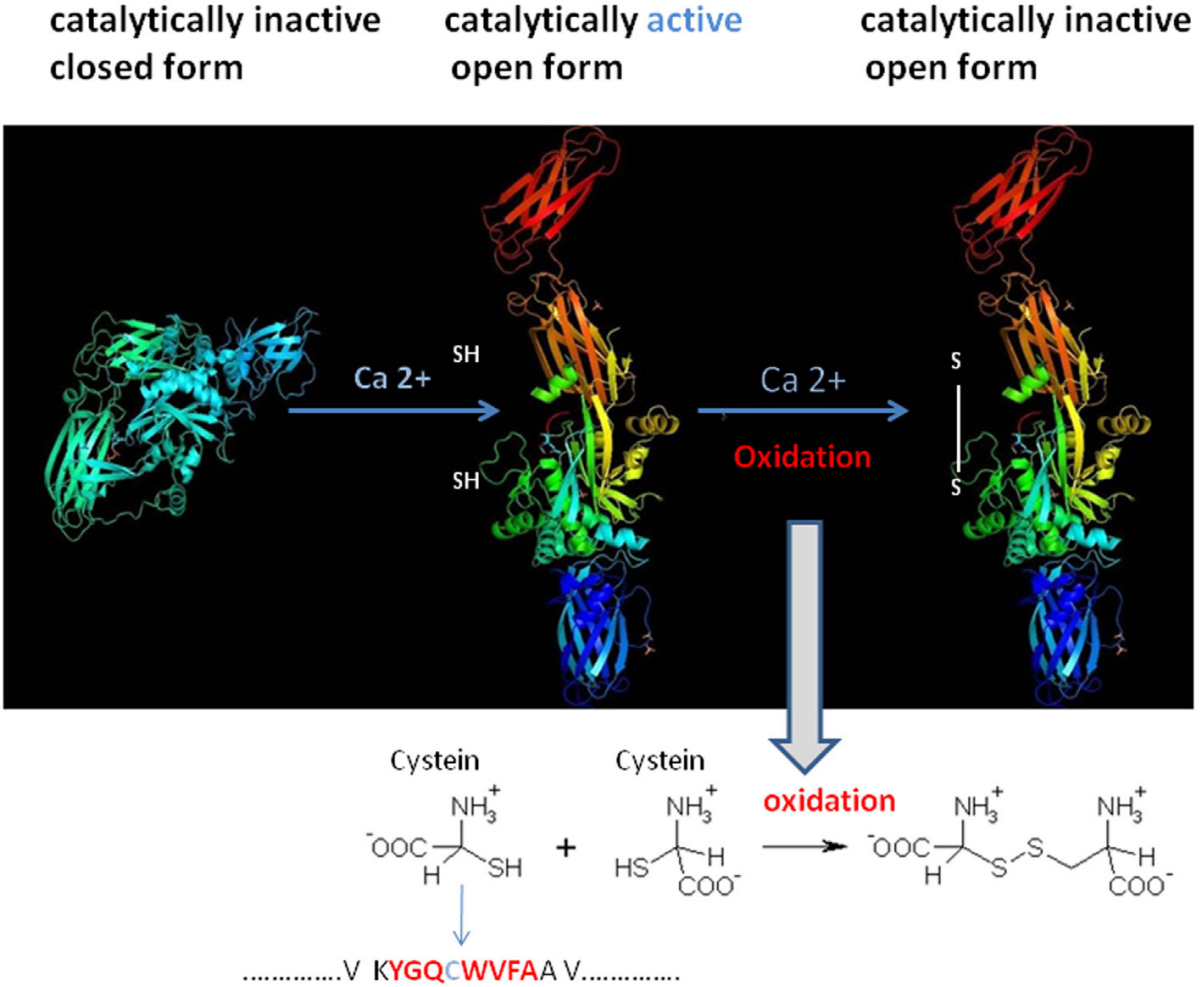
Structural tissue transglutaminase 2 (TG2) conformation influences TG2 catalytic activity. TG2 activity is regulated in cells by reversible conformational changes of the catalytic core site of the protein. The catalytic cystein residue (SH) is part of the conserved motif that is required for the enzymatic activity. Spatial arrangement of the four domains in TG2 leads TG2 domains in an inactive closed compact form. The binding of Ca^2+^ to the catalytic domain of TG2 alters the protein to move domains 3 and 4 away from the catalytic domain, thus making the active site accessible (open, catalytically active). Oxidation of the open/active protein (at the cystein position) results in loss of TG2 activity (open, catalytically inactive form). Modified from PDB, Liu, S. Strop, P. X-ray crystallography structures of tissue transglutaminase.

To understand the Ig–TG2 interaction, it is first necessary to understand how the conformational status of TG2 influences its catalytic activity. TG2 is referred to be a bifunctional enzyme, owing to its ability to catalyze a Ca^2+^-dependent protein cross-linking activity and a Ca^2+^-independent GTP hydrolysis (Figure 3) (16). Cofactors such as GTP/GDP bound to the enzyme render TG2 in a closed compacted form, which obstacle the accessibility of the Ca^2+^-dependent cross-linking site. By contrast, the link with Ca^2+^ alters the TG2 conformation in an open form that expose the catalytic site for a cross-link formation or deamidation based on donor substrates. Thus, cross-linking activity is only observed at high Ca^2+^ concentrations. Since inside living cells Ca^2+^ concentration is low, it is believed that TG2 is predominantly present in a cross-linking-inactive form into the cells, and the cross-linking activity is more present at the cell membrane. However, although there are low GTP and high Ca^2+^ levels in the extracellular milieu the cross-linking activity is not always present (17). To explain this finding, it has proposed a model for which oxidative, nitric oxide, and phosphorylation conditions in the extracellular milieu (e.g., caused by inflammation or aging) point to the inactivation of the TG2 enzyme activity. In addition, proteins present in the extracellular milieu, including fibronectin, osteonectin, and integrins, may also regulate TG2 cross-linking activity. Thus, despite a growing understanding of the biological functions of TG2 and its mechanism of action, many questions remain unanswered like the regulation of its expression and activity in different physio-pathological conditions (17).

**Figure 4:**
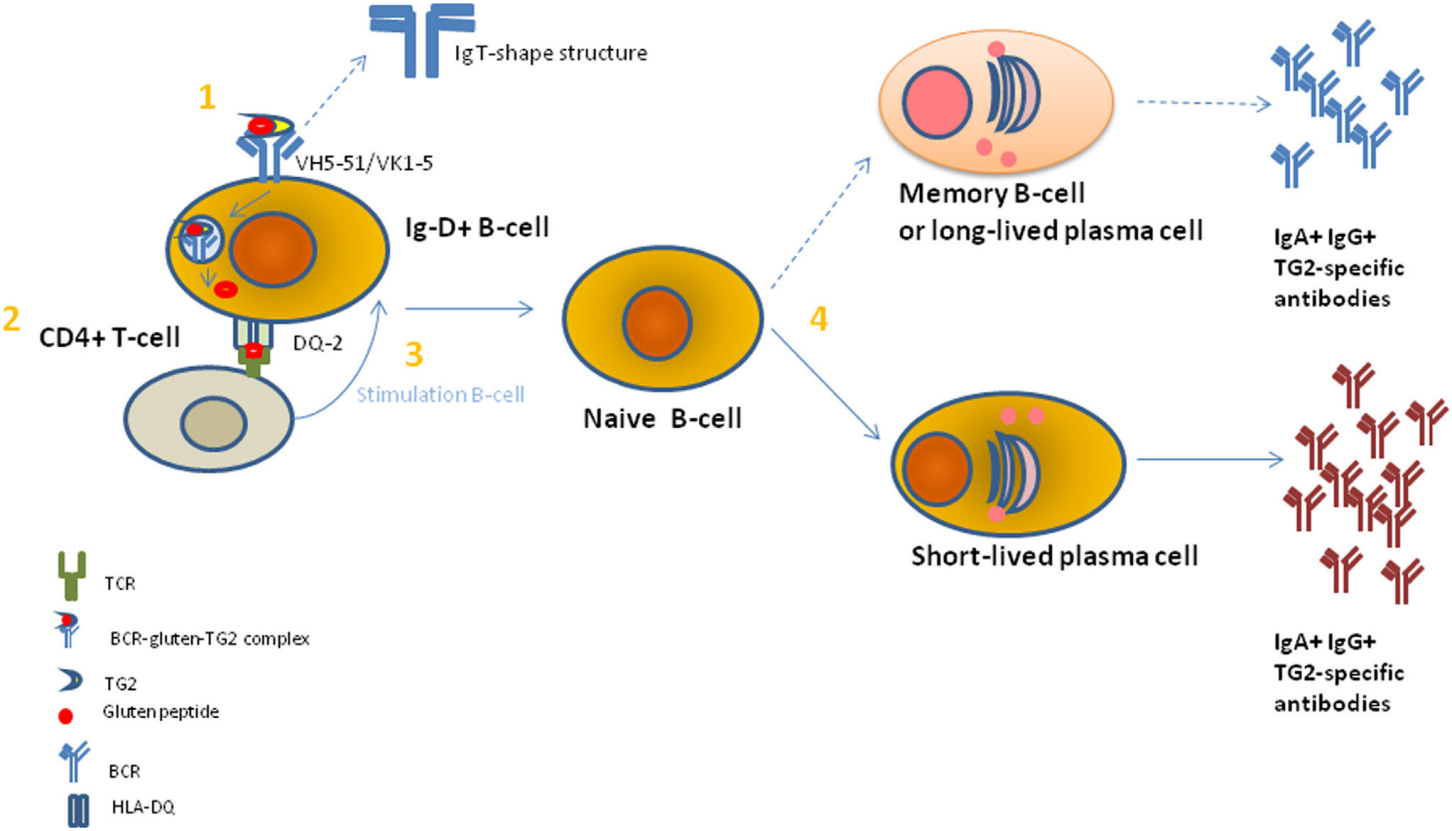
A model to explain the gluten-dependent production of tissue transglutaminase 2 (TG2)-specific antibodies. (1) TG2-gliadin complexes are taken up by a TG2-specific B cell through B-cell receptor (BCR)-mediated endocytosis. BCR shows a restricted use of VH and VK chains of IgD+ istotype. Then, BCR-TG2 cross linked to gliadin peptides complexes are endocytosed and transported to the cellular endosomal compartment where gliadin peptide deaminated by TG2 enzyme were release to bind human leukocyte antigen (HLA)-DQ specific molecules for CD4^+^ T-cell presentation. (2) CD4^+^ T-cell-recognize gliadin peptide presented by HLA-DQ molecule through a specific T-cell receptor (TCR). Correct TCR-peptide interaction causes the signal for B-cell stimulation. (3) B-cell stimulation of IgD+ naïve B-cells leads to the induction of immunoglobulin (Ig) mutation and class switching. (4) Naïve B-cells differentiate in short-lived plasma cells (PCs) (preferentially) and long-lived PCs or memory B cells and release IgA+ and IgG+ TG2-specific antibodies.

To complicate this contest, TG2/gliadin complexes showed to have a key role by a misunderstood mechanism in Ig TG2-autoantibodies production (11). Recently, it is demonstrated that TG2-Igs recognize few distinct conformational epitopes all in the N-terminal part of the TG2 when the enzyme is in an active conformational form induced by the binding with Ca^2+^ (18). Moreover, this region is usually not accessible on cell-surface TG2 (19). By using the X-ray crystallography and small-angle X-ray scattering approaches, the detailed structural interaction between self-antibody and TG2 protein has been evidenced and then confirmed by using the generation of single aminoacid mutations in the TG2 epitope followed by the measure of the protein–protein interaction (20). The single TG2-epitope recognition correlated with specific Ig variable heavy chain (VH)-and light chain (VK) family usage, mainly the IGVH5-51 and the IGKV1-5 gene segments (21). It is known that VH conservation reproduces preservation of aminoacids sequence and structure of the immunoglobulin antigen binding site and, thus, the recognition of a limited set of peptide determinants (22). In the past, several researches including ourselves had used the analysis of Ig V gene usage in target organs and tissues to demonstrate that there is a highly compartmentalized clonal B-cell expansion induced by the presence and recognition of a limited number of Ag/epitopes in several autoimmune and pathological diseases (23). Since TG2 autoantibodies were not only released in the serum but also bound on the membrane of B-cells [B-cell receptor (BCR)], B-cell having this specific BCR was able to present TG2-gliadin complexes to T-cells, which therefore stimulate B-cell maturation and proliferation to plasma cells (PCs) producing TG2-IgM and TG2-IgA (21). The requirement for the presentation of TG2-gliadin to T-cells is intermediated by trafficking of Ag into the B-cells for processing and HLA-presentation, accordantly of the elevated risk for CD in individual-positive for specific HLA-DQ molecules (1). Based on these overall results, it is possible that an epitope within a TG2 domain usually not accessible to recognition by the immune system may be responsible of the autoimmune preservation. Indeed, today the more accredited model to explain the generation of TG2-self-reactive B-cell clones is based on the fact that the TG2/gliadin/Ig complex could be recognized as a new epitope(s), and thus being a “non-self” epitope, this would consent the non-elimination of reactive B-cell clone and then the stimulation for the Ig production. In this contest, the study performed by Iversen and coll (24). Add a new and important information. The authors demonstrated a preferential interaction between the BCR showing an IgD istotype and *IGHV5-51* and *IGKV1-5* gene usage with the TG2 enzyme (24). This preferential interaction was attributed to the higher number of exposed and accessible lysine residues in the hinge region of the IgD istotype, which can adopt a T-shape structure able to incorporate more long peptides than other istotypes, such as IgM, IgG, or IgA (Figure 4). Indeed, the lysine, having a positively charged ε-amino group (a primary amine) that is present on the IgD binding site, induces a strong electrostatic interaction with the TG2 (negative charged) and, thus, consent to efficiently incorporate the enzyme into a high molecular mass complex to form TG2-gliadin–IgD cross-link and then its internalization into the B-cell endosomal compartment. The complex with Ig could protect the TG2 enzyme activity from the endosomal oxidation that would usually block the TG2 activity. Conservation of the TG2 enzymatic activity into the cell lead in the release of more deaminated gliadin peptides, now available for the binding to HLA molecules and presentation to CD4^+^ T-cells. Presentation to T-cells with the appropriate receptor (TCR) for the recognition of HLA-peptides, consequently, induce the stimulation and maturation of IgD^+^ B-cell clones to IgA+ and IgG+ B-cells. This model is in accord with a previous study in active CD, which showed an accumulation of gluten peptides in the intracellular lysosomes particles in association with an oxidative environment and the activation of the TG2 enzyme activity (25). Moreover, TG2 activation into the intestinal cells of patients with CD concomitantly associated with a decreased in the PPAR-gamma-mediated anti-inflammatory activity (PPAR-gamma, anti-inflammatory peroxisome proliferator-activated receptor gamma) (26).

Based on this last knowledge, TG2 autoantibodies and clonal B-cells expansion, and not only T-cells, had been considered important players in CD pathogenesis, leading to the development of novel therapies based on B-cell depletion. However, in practice this approach proved ineffective, probably due to the persistence of long-lived PCs refractory to tested therapies despite the obtained peripheral B-cell depletion (27). Currently, several studies to improve the success of B-cell as well as long-lived plasma-cell depletion in autoimmune diseases are ongoing, e.g., Ref. (28).

Anyway, at present it is clear that the Ig response against TG2 is dependent of TG2 in an active conformation and of a dietary gluten exposure. It is observed that the frequency of TG2-IgA PCs and TG2 memory B-cells decreased, even if they did not completely disappear, in patients who take a gluten-free diet and that the TG2 conformation may be modulated by the release of a large quantity of IFN-ϒ and reactive oxygen species in conditions of stress and inflammation (29). Moreover, the recent structural studies of CD-associated HLA-DQ pockets (i.e., HLA-DQ2.5, HLA-DQ2.2, and HLA-DQ8) had showed that binding of gluten peptides was strictly dependent on the TG2-deamination of the gliadin by introduction of negative charges in proline in the gliadin QXP residues (30). Furthermore, minor differences in HLA-DQ molecules was showed to produce a different affinity for gluten peptides [e.g., HLA-DQ2.5 and HLA-DQ2.2 pockets differed in the binding for Ser in position P3 and, in addition, not all the gluten epitopes were equally well targeted by TG2 (e.g., α- and ω-gliadins are better substrates than γ-gliadin)] (31). Overall, these findings well reflected the difference in the predisposition and response to diverse gluten peptides in different individuals.

In conclusion, TG2 enzyme in CD acted both as a generator of gluten Ag epitopes for T-cells through B-cell/HLA presentation and by the same way as a target for autoantibodies. Both these actions were causally linked and limited to genetically predisposed individuals with the right HLA background. According to this model, resumed in Figure 4, gluten ingestion associated with expression of tissue TG2 enzyme in an active from had a key role in the maintenance of both intestinal mucosal damage and anti TG2-Igs processes.

## Toxic and Immunogenic Gluten Epitopes

Another crucial factor that strongly influences the CD pathogenesis and the clinical manifestation in predisposed individuals is the protein composition introduced with the diet. In wheat, gluten consists of the gliadin and glutenin components. The gliadin proteins can be subdivided into α/β-, γ-, and ω-gliadins; glutenin can be subdivided into high and low molecular weight (HMW; LMW) subunits. All these classes of proteins are encoded by large multigene families (over than 50 genes) encoding similar protein sequences generally with similar biochemical functions and formed by duplication of a single original gene. These genes can be clustered in loci located on a same or on different chromosomes.

On the contrary to other cereals, which are diploid, wheat display a different genome composition ranging from diploid (the earliest cultivated form), to tetraploid and hexaploidy forms. Thus, in a single wheat variety, there exits up to several hundred different prolamines (the cereal storage proteins, such as gliadin having a high proline and Gln content), many of which only differ by a few amino acids. Due to this in wheat there are much more possible peptides that could be toxic (capable of inducing mucosal damage) and/or immunogenic (capable to stimulated HLA-DQ2 and -DQ8 restricted T-cells) compared to other cereals. Each grain has a different set of storage proteins, or different prolamine-like proteins, namely gliadin in wheat, hordeins in barley, secalins in rye, and avenins in oat. Several of toxic epitopes are now identified in wheat and by sequence homology they can be also found in other cereals, such as barley, rye and potentially although in minor quantity in oat (32, 33). Notably, today remains unresolved the debate regarding the potential toxic epitope(s) in oat and the appropriateness of oats in the diet of patients with CD is likely to be controversial (34, 35).

So far, a total of 31 epitopes involved in CD have been identified based on available *in vitro* and *in vivo* evidence of toxicity, the majority of which are derived from gliadins (36, 37). The epitopes contain proline- and Gln-rich heptapeptide PQPQPFP and pentapeptide PQQPY. However, due to the high complexity of the wheat genome and the complex mechanism that induce toxicity in human, multidisciplinary investigations combining several genomic and proteomic approaches are used to dissecting the expression and functionalities of different protein in foods in relation to CD manifestations. The mapping of number of distinct toxic peptides from different varieties of cereals is still incomplete and is a matter of on-going discussion (38).

**Figure 5:**
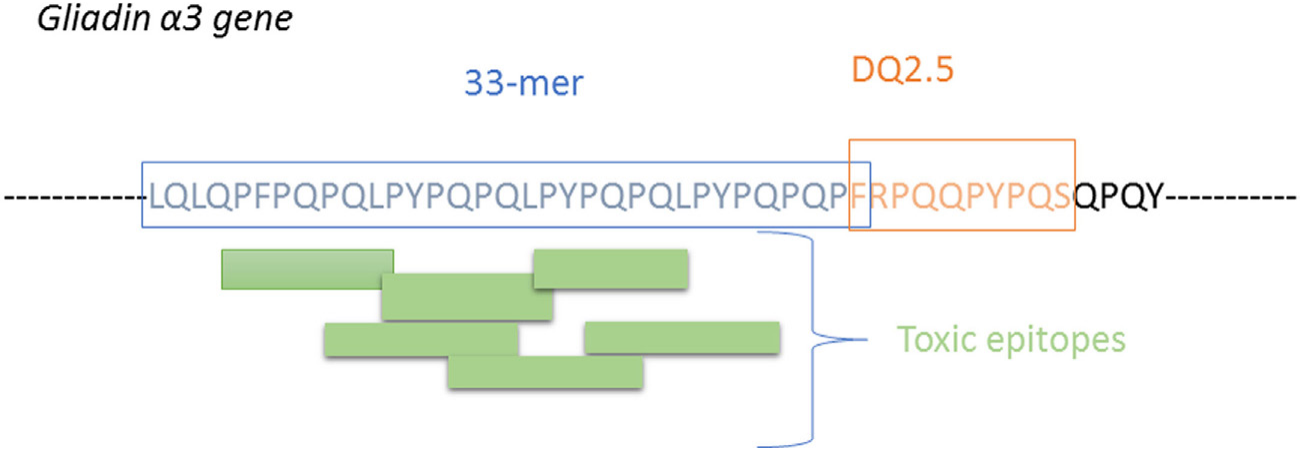
Illustration of the 33-mer proteolytically resistant site presnt on α-gliain, showing the T-cells sites and the additional immune peptide overlapping the 33-mer peptide.

In CD, reactive T-cells also recognize the toxic epitopes (immunogenic epitope) but with a greater extent when specific Gln are deamidated by the tissue TG2 enzyme. The recognition is depended of HLA-DQ molecules and TCR sequence. HLA-DQ2 is the predominant molecule in CD (over 90% of CD patients) It is now well accepted that the major gluten protein involved in CD is the α-gliadin isoform having the main 33-mer immunogenic fragment comprising six overlapping DQ2-restricted toxic epitopes (blocks of PFPPQQ and PYPQPQ) and an additional DQ2-restricted epitope which partially overlaps with the 33-mer peptide (4, 30, 37) (Figure 5).

Recent advances in proteomic tools facilitated examination of HLA-peptidomes leading to a more comprehensive quantitative and comparative analysis of HLA-peptide complexes based on specific HLA-DQ haplotypes. Briefly, the procedure consists of the isolation of the HLA-DQ/peptide complexes by using antibody DQ-specific immunoprecipitation and then elution and purification of the peptides by reverse phase chromatography and mass/mass fingerprinting identification (31). By using this procedure authors demonstrated that the repertoire of eluted peptides were unique for each of the DQ molecule tested (i.e., the serine at position P3 of the binding frame bind with a higher affinity the DQ2.2 molecule compared with the alanine in the same position, while an opposite tendency was found by using the DQ2.5 molecule). Thus, a single amino acid was instrumental for a stable HLA-peptide complex, an indispensable condition for an efficient peptide presentation to T-cell, thus demonstrating the different CD risk for patients carrying different HLA-DQ molecules. Based on the reported sequence motifs several programs have been developed, e.g., BIOPEP (www.uwm.edu.pl/biochemia), for *in silico* assessment of potential amino acid and the surrounding amino acid sequences with high degree of identity to toxic peptides. This would consent to identify and characterize the essential motifs associated with CD, their variants and their abundance in the cereal.

A deepening of these studies is useful to better understand the effect of wheat varieties on immune cell stimulation and to resolve the question of whether the environment has an effect on the worldwide increase in CD incidence. Nowadays, there is unmistakable evidence that beyond α- and β-gliadins other proteins also contain T-cell stimulatory peptides. It is widely accepted that high concentration of Gln and, especially, proline prevents the complete degradation by human gastric and pancreatic enzymes, thus resulting in the build up of oligopeptides in the small intestine that are resistant to further proteolysis. Indeed long proteolytically resistant fragments containing multivalent peptides were found, e.g., in α-gliadin, γ-gliadin, glutenin, hordein, secalin, while in contrast, proteins with limited T cell antigenicity present for example in bovine myoglobin, chicken ovalbumin, and lactoglobulin producted peptides no longer than 10 amino acids and only avenin and bovine casein were demonstrated to produced digestion fragments of 8–10 residues (39). It is possible that the rapid intestinal brush-border membrane proteolysis of peptides shorter than 10 amino acids, the potentially antigenic peptides released from these proteins are likely to be rapidly destroyed. Notably, avenin fragments long to 10 residues and recognized by intestinal T cells was found in oats-intolerant CD patients, thus supporting the above reported hypothesis (40).

Overall these results clearly reinforce the notion that protease stability and immunotoxicity of gluten are intimately correlated. Apply to this with the aim to reduce the antigenicity of toxic peptides several studies investigating protein engineering of wheat and potential therapeutic enzymes that could be introduced with diet are ongoing. Today, few potential candidate enzymes have been tested, most involving prolyl endopeptidase, an enzyme that in human is encoded by the PREP gene. Prolyl endopeptidase is the only human enzyme known to be able to cleave the 33-mer and the 26-mer proline-rich gluten peptides. Efficient degradation of gluten by microbe prolyl endopeptidase are object of several studies, including cultivable oral microbiome (41, 42), however, most of the enzymes studied have limitations as they are irreversibly inactivated by pepsin and acidic pH, both present in the stomach. Two modified enzyme options in advanced testing and resistant to gastric inactivation are a prolyl endoprotease from *Aspergillus niger* [AN-PEP; (43, 44)], and a mixture of two gluten-specific recombinant proteases, the ALV003 (45). Both these enzymes appear to be effective, well tolerated, and clinical data are emerging that demonstrate the enzymes can attenuate intestinal injury (46, 47).

## The Microbiome and CD

Gluten has immune effects in a given individual at a certain time of his live while he had consumed gluten since childhood. In the last decades, it is proposed that gut microbe(s) present in CD patients could help as a trigger to initiate the CD4^+^-T-cells response and induced loss of oral tolerance to the gluten.

The first hypothesis that connects microbes to CD had proposed an immunological cross-reactivity between antigenic determinants shared by the viral protein and gliadins (48). Several epidemiological associations between viral infections, mainly intestinal adenovirus and enterovirus (e.g., rotavirus), and CD, have been reported (49, 50). However, today results of the above reported study remain controversial (51).

A similarity between the adhesive protein (Hwp1) of the yeast *Candida albicans* and two known CD-related gliadin T-cell epitopes has been reported as a substrate for mammalian transglutaminase (52) leading to the hypothesis that *Candida* may be a trigger for CD (53). More recently, characterization of the gastrointestinal microbiome identified *Candida* as a dominant fungal genera in CD patients (54, 55) and, moreover, serological cross-reactivity between *Candida* Ag and gliadin was evidenced (56, 57), hence, adding another link between *C. albicans* infection and CD.

Given the limitations of these studies, we cannot conclude that there is a relationship of cause and effect between *Candida* and CD; nonetheless, these results added important observations to support the hypothesis.

By the same time, there is a growing body of evidence to suggest that the composition of the overall intestinal microbiome is associated with a number of chronic gut diseases, for example, functional dyspepsia and irritable bowel syndrome (58). The composition of the gastrointestinal microbiome of susceptible individuals with CD has also been investigated in a number of studies (59–61). Indeed, although the mechanism is still unknown patients with the HLA-DQ2 haplotype associated with CD susceptibility show a selection for a gut microbiota composition with a high proportion of gut firmicutes and protebacteria and a reduction in a bifidobacteria (62, 63). Furthermore, several studies based on different animal models have also indicated an association between certain major histocompatibility complex polymorphisms and fecal microbiota composition (64–67) as well as an association in respective subjects between the microbiota profile and the amount of blood DNA methylation (68). Thus, several studies suggest that there is an association between genetic and epigenetic factors associated and a distinct microbiome in CD patients. Based on this knowledge and through new proteomic approaches, it is now possible to identify microorganisms present in the gut of a definite individual and verify their potential efficiency to degrade the gluten peptides (41).

Analysis of the microbiome composition could be also useful to analyze part of the environmental factors that may play a role in CD development. A contribution in this direction was given by metabolomic approach. Nuclear magnetic resonance of urine and serum samples of CD patients demonstrated a metabolic signature of CD patients, which may revert to a “healthy” metabolomic profile after 12 months from beginning a strict gluten-free diet (69), and in addition a bacterial dysbiosis was also evidenced both in fecal and salivary samples of patients with CD (70, 71). In particular, metabolomic analyses showed a significant higher level of short-chain fatty acids (SCFAs) in CD patients compared to the controls (72). SCFAs may reflect the metabolic activity of intestinal microbiota, which could be modified by dietary and environmental changes (73). Bacterial cross-feeding has a huge impact on the final balance of SCFA production and the efficient exploitation of the substrates that reach the human gut (e.g., *Bifidobacterium* species, *Bacteroides uniformis*, and *Escherichia coli*). These mechanisms consist either in the utilization of end products from the metabolism of a given microorganism by another one, called metabolic cross-feeding and/or the utilization by one microorganism of the energy-rich complex CHO breakdown products formed by another one, called substrate cross-feeding. SCFAs are important anions in the colonic lumen not only to provide the major source of energy for colonocytes but also to exert anti-inflammatory effects, which influence various functions of the intestinal tract and the growth of known pathogens such as *Pseudomonas* (74). However, the significance of SCFA imbalance in CD required to be validated in perspective studies and the bacteria species specifically associated with SCFA imbalance ask to be identified.

Nonetheless, overall the results confirmed that a gut microflora may be altered and associated with the clinical manifestation of CD and that a gluten-free diet could not completely restore the “normal” intestinal microbiota in CD patients.

## Conclusion

Overall, new insight into the pathogenesis of CD is shifting toward an important role of B-cells in addition to CD4^+^ T-cells. In addition, overall ongoing knowledge regarding characterization of the host microbiome and the better definition of toxic peptides at molecular and structural level had added new and relevant information to better understand the mechanism that could initiate the CD4^+^-T-cells response and induce the loss of oral tolerance to the gluten. Overall, these results are of potential consequences for therapeutic approaches targeting on plasma-cell depletion and on modified propyl-endopeptidase enzymes. The employment of proteomic techniques in the study of CD is only in its initial stage and rapid technological development of proteomic platform supported by classical genetic and epigenetic methodologies will lead to new discoveries, which will improve the knowledge of CD pathogenesis. The hope is that through advances in technical approaches involving culture-independent molecular methods and high-throughput analysis comprehensive studies of changes in the resident intestinal microbiota and host immune system reactivity will provide new vision of the molecular mechanism for sustaining CD.

## Consent for Publication

Results/data/figures in this manuscript have not been published elsewhere, nor are they under consideration by another publisher.

## Availability of Data and Material

Data are freely available for the readers.

## Author Contributions

VR, RM, and RC wrote and edited the manuscript, VR performed figures and critically revised the manuscript. All authors read and approved the final manuscript.

## Conflict of Interest Statement

The authors declare that the research was conducted in the absence of any commercial or financial relationships that could be construed as a potential conflict of interest.

## Abbreviations

CD, celiac disease; HLA, human leukocyte antigen; Ig, immunoglobulin; RNA-seq, next-generation RNA-sequencing; IFN-γ, interferon-gamma; TG2, tissue transglutaminase 2; IL, interleukin; MHCs, major histocompatibility complexes; Ab, autoantibody; BCR, B-cell receptor; CDR, complementary regions; IVH, IVK, variable immunoglobulin heavy and kappa chains, respectively; PAD, peptidyl arginine deaminases; PPAR, peroxisome proliferator-activated receptor; PCs, plasma cells; TCRs, T-cell receptors; SCFAs, short-chain fatty acids; NMR, nuclear magnetic resonance.
